# Novel Mechanism of the L‐Type Voltage‐Gated Channels/Calpains Axis in Influenza A Virus‐Induced Autophagosome Activity

**DOI:** 10.1002/jmv.70631

**Published:** 2025-10-04

**Authors:** Hannah Murphy, Hinh Ly

**Affiliations:** ^1^ Department of Veterinary and Biomedical Sciences, College of Veterinary Medicine University of Minnesota St. Paul Minnesota USA; ^2^ Department of Math, Science, and Technology University of Minnesota Crookston Minnesota USA

**Keywords:** autophagosome, autophagy, influenza A virus (IAV), l‐type voltage‐gated channels

Seasonal influenza viruses can cause up to 1 billion human infections and can result in 290 000–650 000 deaths annually worldwide [1]. The global influenza burden varies widely due to a complex interplay of factors, such as viral strain characteristics (i.e., transmissibility, severity, and antigenic drift), seasonal patterns (i.e., temperature, humidity, and human behavior), vaccine effectiveness, and immunization coverage [2, 3]. While vaccines are the most effective means for preventing seasonal influenza A virus (IAV) infections in healthy adults, they provide suboptimal protection for high‐risk groups and can be ineffective when antigenic predictions in seasonal vaccine formulation happen to be mismatched with the circulating or emerging virus strains [4]. In addition to vaccines, antivirals are widely used to control IAV infections, with the main antiviral classes targeting the viral neuraminidase and cap‐dependent endonuclease, whereas the M2 inhibitors not being recommended due to the rapid emergent of viral resistance [5, 6]. The circulating IAV strains continuously develop resistance to all available forms of antivirals that highlight the need for new approaches against IAV infection.

The recently published article by Tian et al., entitled “Influenza A Virus Induces Autophagosome by Inhibiting LTCC/Calpain 2/LC3A Signaling to Promote Viral Replication” in the Journal of Medical Virology [7], investigated a novel mechanism of the l‐type voltage‐gated channels (LTCC)/calpains axis in IAV‐induced autophagosome activity. Using the PR8 (H1N1) IAV isolate, the authors demonstrated, through a series of well‐thought‐out experiments, that IAV infection reduces LTCC‐mediated Ca^2+^ influx; in human lung adenocarcinoma A549 cells, Cav1.3 is the predominant LTCC isoform, and its knockdown (KD) phenocopies LTCC blockade, consistent with suppression of Cav1.3 activity during IAV infection.

Briefly summarized, the authors demonstrated that under normal conditions (Figure 1, left), Cav1.3 induces Ca^2+^ influx, activating calpain‐2, which cleaves LC3A and maintains normal levels of autophagosomes. However, in PR8 (H1N1) IAV‐infected cells (right), Cav1.3 is suppressed, which decreases the Ca^2+^ influx, leading to calpain‐2 being inactivated. LC3A is therefore being left uncleaved, and autophagosome accumulation occurs concurrently with IAV blocking the fusion of autophagosomes and lysosomes into autolysosomes. The authors showed that the accumulation of autophagosomes could promote IAV viral replication in human lung adenocarcinoma A549 cells. Under physiological conditions, LC3A is primed at the C‐terminus by ATG4 proteases for subsequent lipidation [6]. This study [7] extended those findings by showing that calpain‐2 can cleave LC3A (aa112‐118) to suppress autophagosome formation. The authors also showed that PR8 (H1N1) IAV infection of A549 cells suppresses the Cav1.3‐calpain‐2 axis, which results in LC3A‐dependent autophagosome accumulation without lysosomal fusion, thus promoting viral replication (Figure 1, right).

**Figure 1:**
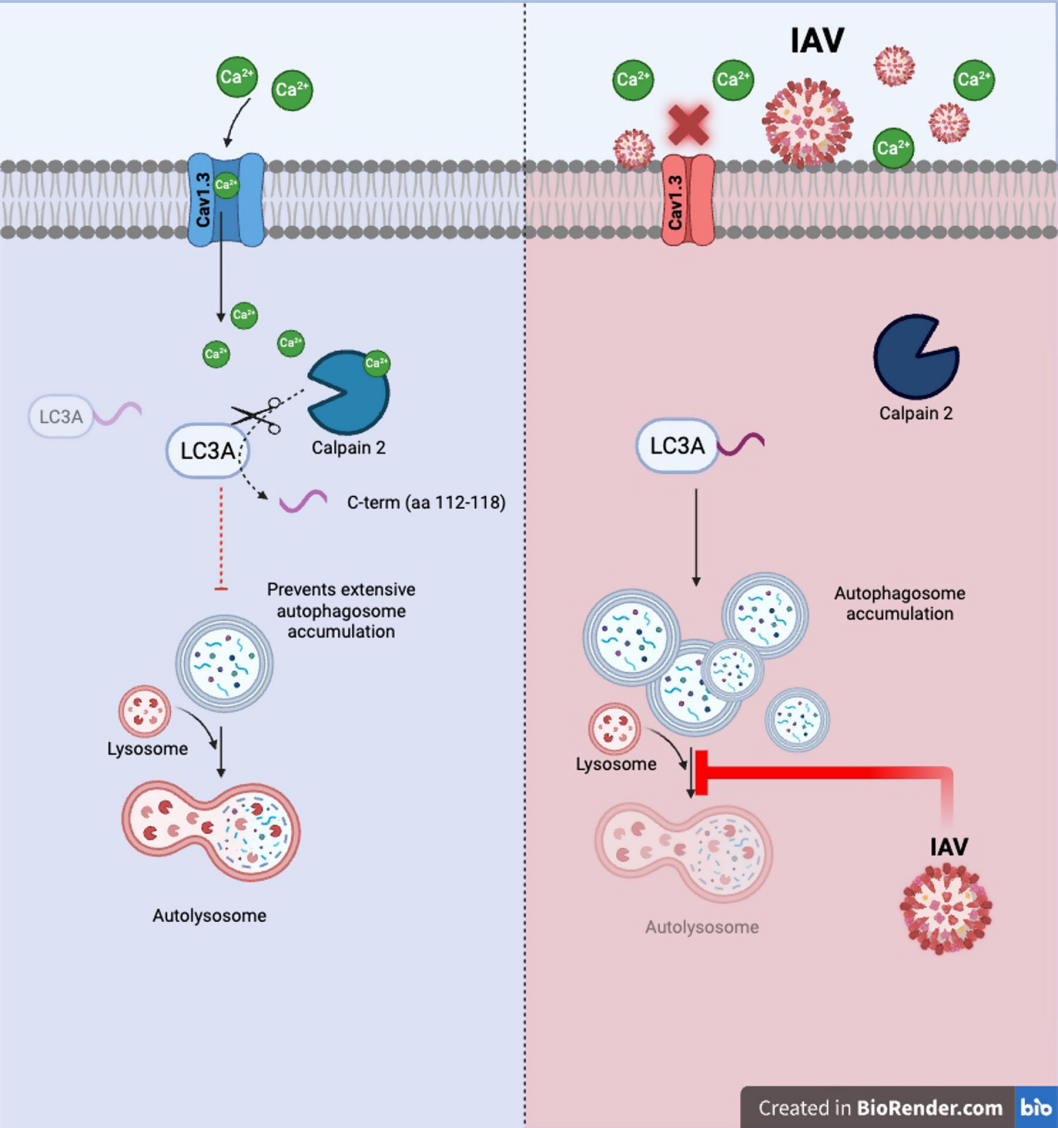
Proposed mechanism of proviral IAV regulation of LTCC (Cav1.3)/calpain 2/LC3A signaling pathway. In healthy cells (left), Cav1.3 induces Ca^2+^ influx, activating calpain 2, which cleaves LC3A and maintains normal levels of autophagosomes. However, in IAV‐infected cells (right), Cav1.3 is suppressed, which decreases the Ca^2+^ influx, leading to calpain 2 being inactivated. LC3A is therefore being left uncleaved, and autophagosome accumulation occurs concurrently with IAV blocking the fusion of autophagosomes and lysosomes into autolysosomes. Accumulation of autophagosomes promotes IAV viral replication in human lung adenocarcinoma A549 cells. Modified figure from Tian et al., 2025 [7], which is made using BioRender.

Experimentally, the authors used PR8 (H1N1) IAV‐infected cells and pharmacological agonists and antagonists to show that cellular autophagy is inhibited by IAV at a late step in the autophagic pathway, that is, the fusion of autophagosomes and lysosomes. By using live‐cell Ca^2+^ imaging throughout the virus infection cycle, they showed a persistent level of decline in cytoplasmic Ca^2+^ levels, suggesting an upstream ion‐channel regulation. When pharmacologic activation of LTCCs was performed, Ca^2+^ levels were partially restored, reducing autophagosome formation and therefore hindering PR8 (H1N1) IAV replication. On the contrary, LTCC inhibition yielded the opposite effects, indicating that LTCCs sit upstream of the IAV‐induced block of cellular autophagy. Knockdown (KD) of Cav1.3, an LTCC isoform, resulted in increased autophagosome formation and enhanced viral replication, which led the authors to investigate the role of calpains, which are downstream targets of LTCC and are known to be important in autophagy. KD of calpain‐2, but not calpain‐1, significantly upregulated the expressions of the cellular LC3‐II and the viral M1 gene, which is an abundantly expressed IAV protein and can be used as a marker for viral replication, thereby leading the authors to conclude that calpain‐2 is mediating the effect of LTCC/Cav1.3 on autophagosome formation (i.e., by cleaving LC3), as well as on PR8 (H1N1) IAV replication. Lastly, the authors used a combination of in‐silico site prediction with tag‐orientation assays and a cleavage‐resistant mutant to localize LC3A cleavage to the C‐terminus (aa 112‐118), which suppresses autophagosome formation.

Traditionally, autophagy has been viewed as an antiviral defense pathway (e.g., via antigen presentation, xenophagy) [8]; however, many viruses can manipulate the host autophagy machinery to enhance their own replication [9, 10]. IAV is a clear example as it can drive autophagosome accumulation and blocks autolysosome formation, to benefit viral replication [11]. However, pharmacologic activation of LTCC via the BAY K8644 compound restores Ca^2+^ signaling, attenuates autophagosome build‐up, and suppresses IAV replication in vitro. As such, this study [7] highlights a potential practical pharmacological intervention to functionally separate the autophagy mechanism as “friend” versus “foe.” Beyond understanding the underlying mechanism, the translational capabilities are numerous not only for IAV but also for many other human respiratory viruses that mirror IAV's ability to exploit the increased autophagosome accumulation level and to stall autophagosome‐lysosome fusion. Some of these examples include SARS‐CoV‐2 (e.g., ORF3a blocking autophagosome and/or amphisome fusion with lysosomes) [12], human parainfluenza virus type 3 (HPIV3) (e.g., HPIV3 phosphoprotein prevents host SNARE proteins from mediating autophagosome‐lysosome fusion) [13], and respiratory syncytial virus (RSV) (e.g., RSV inhibits autophagosome‐lysosome fusion but IL‐22 restores cellular autophagy) [14].

While the authors of the current study have described the involvement of the LTCC‐calpain‐LC3A axis in IAV infection [7], several mechanistic details remain uncharacterized, specifically how IAV selectively downregulates Cav1.3. Downregulation of Cav1.3 by IAV may occur at various levels, including transcriptional/translational control, posttranslational removal, or modulation of the l‐type voltage‐gated channels. Experiments to carefully discriminate between the possible mechanisms and levels of Cav1.3 downregulation will help determine whether virus‐induced LTCC agonism is a result of a trafficking deficit or a gating block. While the authors have provided compelling evidence via Western blotting analysis to support the cleavage of LC3A by calpain‐2, adding biochemical analysis, for example, cleavage mapping by mass spectrometry, would clarify the mechanism further.

Additionally, expanding the in vivo mouse lung transmission electron microscopy data to include some biological readouts, such as viral titrations, animal survival curves, and histopathological analysis, would further strengthen the conclusions. It is important to also note that this study utilized a laboratory strain of IAV (PR8 H1N1) and at a relatively high multiplicity of infection (MOI = 2), which can limit its generalizability. Future confirmation studies in primary human airway cell cultures and using currently circulating IAV isolates at lower (multi‐cycle) MOIs could help address physiologically relevant concerns. Additional studies of the specifics of which LC3 paralog is most important in airway epithelium and whether there are any redundant mechanisms present in this LTCC‐calpain‐LC3A axis need to be done. Finally, while the M2 protein of IAV has been shown to block autophagosome‐lysosome fusion [15] and that it contains an LC3‐interacting region that sequesters LC3 to virus‐controlled membranes [16], the specific viral protein(s) responsible for the downregulation of Cav1.3 remains to be identified.

Overall, the current study [7] provides some compelling evidence for a model where PR8 (H1N1) IAV can manipulate the LTCC/calpain‐2/LC3A pathway to enhance its own replication. Future studies are needed to characterize the level (i.e., gene regulation, trafficking, or gating) at which Cav1.3 is being downregulated and to pinpoint the specific viral protein(s) responsible, which will help clarify the mechanism and increase the translatability factor of the study.

## Ethics Statement

The authors have nothing to report.

## Conflicts of Interest

The authors declare no conflicts of interest.

## Data Availability

The authors have nothing to report.
